# Type 1 Cryoglobulinemic Glomerulonephritis With IgG2-Kappa Arising From Monoclonal Gammopathy of Undetermined Significance: A Case Report and Literature Review

**DOI:** 10.7759/cureus.88430

**Published:** 2025-07-21

**Authors:** Zainulabdeen S Al-saedi, Lina Alatta, Mohammed A Miqdad, Hasan Hulwi, Oscar Rodriguez, Yihe Yang, Sheng Kuo

**Affiliations:** 1 Medicine/Nephrology, NewYork-Presbyterian Queens, New York, USA; 2 Nephrology, NewYork-Presbyterian Queens, New York, USA; 3 Research, Michigan State University, East Lansing, USA; 4 Nephrology, NewYork-Presbyterian, New York, USA; 5 Internal Medicine/Nephrology, NewYork-Presbyterian Queens, New York, USA; 6 Pathology and Laboratory Medicine, Weill Cornell Medicine, New York, USA

**Keywords:** cryoglobulinemia vasculitis, igg2-kappa, monoclonal gammopathy of renal significance (mgrs), monoclonal gammopathy of undetermined significance (mgus), type 1 cryoglobulinemic glomerulonephritis

## Abstract

This case report describes a rare presentation of Type 1 Cryoglobulinemic Glomerulonephritis due to IgG2 kappa monoclonal gammopathy in a 74-year-old man with a history of Monoclonal Gammopathy of Undetermined Significance (MGUS), classifiable under Monoclonal Gammopathy of Renal Significance (MGRS). The patient presented with acute kidney injury, hypertensive urgency, and a migratory rash. Kidney biopsy revealed glomerulitis with IgG2-kappa deposition. Pseudothrombi of IgG2-kappa paraprotein were also present within glomerular capillary and arteriolar lumens, accompanied by vasculitis. Although initial outpatient management focused on MGUS, rapid disease progression necessitated aggressive therapy, including high-dose corticosteroids, plasmapheresis, and hemodialysis. The patient’s renal function improved following treatment, emphasizing the need for early recognition and timely intervention in cryoglobulinemia-related kidney disease. This case highlights the importance of kidney biopsy in diagnosing MGRS and underscores the role of targeted therapies in complex cases, particularly clone-directed treatment following initial clinical stabilization.

## Introduction

Monoclonal Gammopathy of Undetermined Significance (MGUS) is a plasma cell disorder characterized by the presence of an abnormal monoclonal protein in the blood without evidence of malignant disease and end-organ injury. It is often an asymptomatic condition discovered incidentally during routine laboratory testing [1]. MGUS is frequently detected in older individuals and typically progresses to more serious diseases such as multiple myeloma, lymphoma, or other plasma cell-related malignancies in a small percentage of patients [2]. It remained unpredictable regarding who will progress and how the progression will manifest clinically. Renal involvement may be a complication of MGUS, as monoclonal immunoglobulins can deposit in the kidneys, leading to a spectrum of disorders such as light chain deposition disease, cast nephropathy, cryoglobulinemia (CG), proliferative glomerulonephritis with monoclonal immunoglobulin deposits (PGNMID), and C3 glomerulopathy [3]. We report a case of rapidly progressive glomerulonephritis in a patient with MGUS who developed biopsy-proven IgG2-kappa type 1 cryoglobulinemia despite close hematology follow-up. A brief review of the literature is also included.

## Case presentation

A 74-year-old man presented with a past medical history of hypertension, anemia, MGUS, and rheumatoid arthritis controlled on methotrexate and sulfasalazine. His outpatient hematological evaluation was significant for hemoglobin of 10.2 g/dL associated with IgG kappa monoclonal paraproteinemia, serum free kappa/lambda ratio of 4.34, and 5% of plasma cell on bone marrow biopsy. He did not have hypercalcemia, renal dysfunction, or proteinuria. Bone marrow flow cytometry was negative for monoclonal abnormality. The plan was for conservative monitoring without the need for active treatment.

A little over a month after his last hematology follow-up, he presented to the emergency department with shortness of breath and chest pain. His blood pressure was 200/102 mmHg, and he had clinical signs of volume overload, including lower extremity edema. He reported a tender rash, which first appeared a few weeks prior to his acute illness that started on his left foot and later migrated to the left leg. Initial laboratory findings are summarized in Table 1.

**Table 1:** Relevant Initial Laboratory findings WBC: White blood cells, Hbg: Hemoglobin, MCV: Mean Cell Volume, MCH: Mean Cell Hemoglobin, Plat: Platelets, Na: Sodium, K: Potassium, Cl: Chloride, CO2: Bicarbonate, BUN: Blood Urea Nitrogen, Cr: Creatinine, Glu: Glucose, AG: Anion Gap, Ca: Calcium, Pr/Cr: Protein Creatinine ratio, R.R: Reference range

Complete Blood Count	
Labs	Value (R.R)
WBC	7.76 (4.8-10.8 x 10^3^/uL)
Hgb	11.7 (13.3-17.7 g/dL)
MCV	85.6 (80-100 fL)
MCH	29.6 (26-34 pg)
Plat	269 (150-400 x 10^3^/uL)
Metabolic Panel	
Labs	Value (R.R)
Na	128 (136-145 mmol/L)
K	7.3 (3.5-5.1 mmol/L)
Cl	95 (98-107 mmol/L)
CO2	14 (22-29 mmol/L)
BUN	89.8 (8-23 mg/dL)
Cr	10.51 (0.67-1.17 mg/dL)
Glu	101 (74-107 mg/dL)
AG	19 (5-17)
Ca	7.5 (8.6-10.4 mg/dL)
Urinalysis	
Labs	Value (R.R)
RBC	9 (0-2/HPF)
WBC	1 (0-3/HPF)
PH	6 (5-8)
Protein	100 (Negative/Trace mg/dL)
Blood	Trace (Negative)
Urine Pr/Cr	1,539.1 mg/g (<11 mg/g)

CT abdomen without contrast demonstrated diffuse predominantly subcentimeter lytic lesions throughout the osseous structures, and the kidneys were unremarkable; however, PET imaging showed no suspicious FDG-avid lytic lesions. Additional serologies are shown in Table 2.

**Table 2:** Relevant serological tests C3, C4: Complement 3 and 4, ANA: Antinuclear Antibody, Anti-SM: Anti-Smith, CCP: Anti-Cyclic Citrullinated antibody, RF: Rheumatoid factor, MPO: Myeloperoxidase, PR3: Proteinase 3, HBSAG: Hepatitis B surface antigen, HCV Ab: Hepatitis C virus antibody, AMA: Anti-mitochondrial antibody, R.R: Reference Range.

Lab	Value (R.R)
Kappa (K)	1019.69 (0.33-1.94 mg/dL)
Lambda (L)	66.96 (0.57-2.63 mg/dL)
Free K/L	15.23 (0.26-1.65)
C3	121 (82-185 mg/dL)
C4	30 (15-53 mg/dL)
ANA	1:80 (<1:80)
Anti-SM	<1 (<1:Negative)
CCP	<16 (<20: negative)
RF	<10 (0-14 IU/mL)
MPO	0 (0-19 AU/mL)
PR3	1 (0-19 AU/mL)
HBSAG	negative
HCV Ab	negative
AMA	1:80

Bronchoalveolar lavage was negative for malignant cells. Bone marrow biopsy showed ~5% patchy plasma cell infiltrate; flow cytometry identified an abnormal plasma cell population with 53.1% CD138-positive cells. Conventional karyotyping showed no clonal chromosomal abnormalities; however, FISH detected trisomy 21, consistent with incidental mosaicism and unrelated to the plasma cell clone.

Kidney biopsy was performed with finding of cryoglobulinemic vasculitis. Cryoglobulin was detected in the serum; however, quantitative measurement was not performed.

He was initiated on thrice-weekly dialysis starting on hospital day 2, and plasmapheresis was commenced after the kidney biopsy, with a total of five sessions using albumin as replacement fluid. He was subsequently transferred to the Liquid Tumor Center for further management. At the time of this manuscript, he is off hemodialysis, which was discontinued after the final session on hospital day 35, with a serum creatinine of approximately 1 mg/dL. Ongoing clone-directed therapy began with a bortezomib-based regimen (DCyBorD), leading to partial remission and resolution of azotemia and proteinuria, followed by daratumumab, carfilzomib, lenalidomide, and dexamethasone (DKRd) therapy.

Renal pathology

Microscopic Description

**Figure 1:**
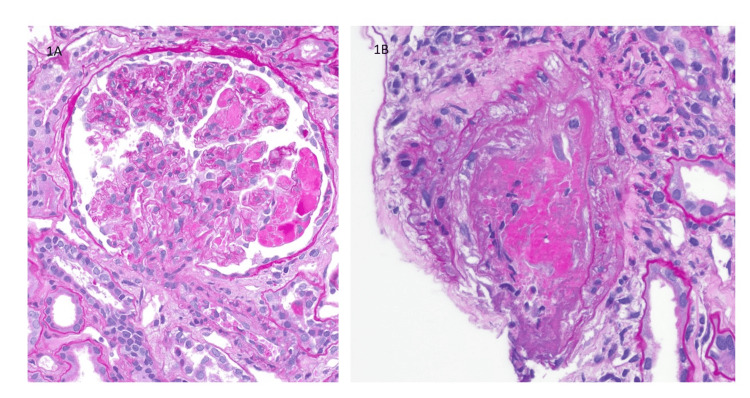
(A) Numerous hyaline "microthrombi" are found in the glomerular capillaries. (PAS stain, magnification: 400x). (B) Hyaline "pseudothrombi" are present within the lumina of occasional small arteries. Affected arteries show focal vasculitis with mononuclear inflammatory cell infiltration. (PAS stain, magnification: 200x)

**Figure 2:**
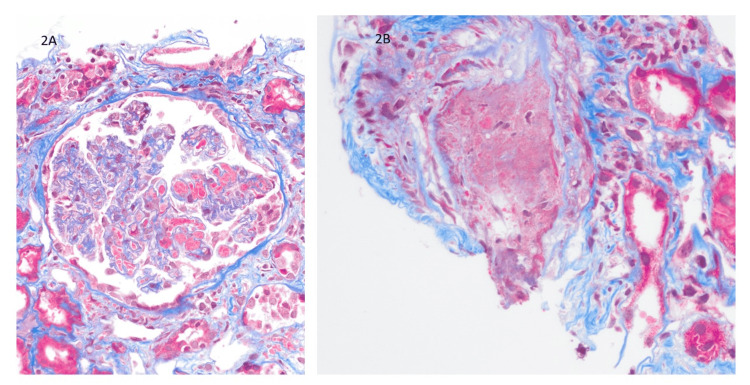
(A) Numerous hyaline "microthrombi" are found in the glomerular capillaries. (trichrome stain, magnification: 400x). (B) Hyaline "pseudothrombi" are present within the lumina of occasional small arteries. Affected arteries show focal vasculitis with mononuclear inflammatory cell infiltration. (trichrome stain, magnification: 200x)

The sample submitted for light microscopy biopsy consists of 39 glomeruli, of which nine are globally sclerosed. The kidney biopsy sample reveals a membranoproliferative pattern of glomerular injury. Numerous hyaline "microthrombi" are found in the glomerular capillaries. About 20-30% of the cortex exhibits tubular atrophy and interstitial fibrosis, with moderate sclerosis of both arteries and arterioles. Hyaline "pseudothrombi" are present within the lumina of occasional small arteries. Affected arteries show focal vasculitis with mononuclear inflammatory cell infiltration (Figure 1 and Figure 2).

Immunofluorescence Microscopy

**Figure 3:**
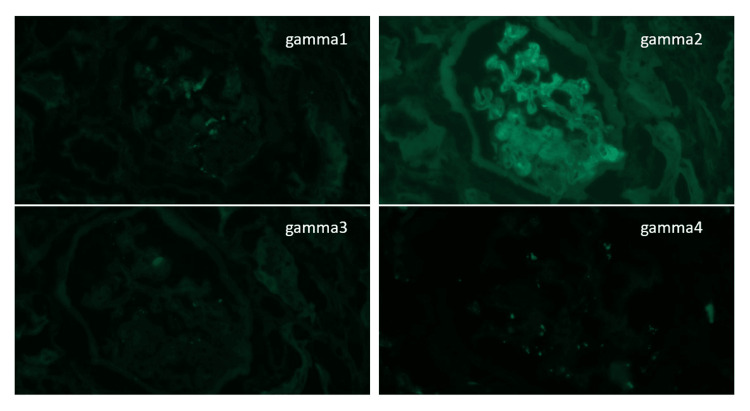
Immunofluorescence (IF) on frozen section: Reactivity for IgG subtypes reveals IgG2 restriction.

**Figure 4:**
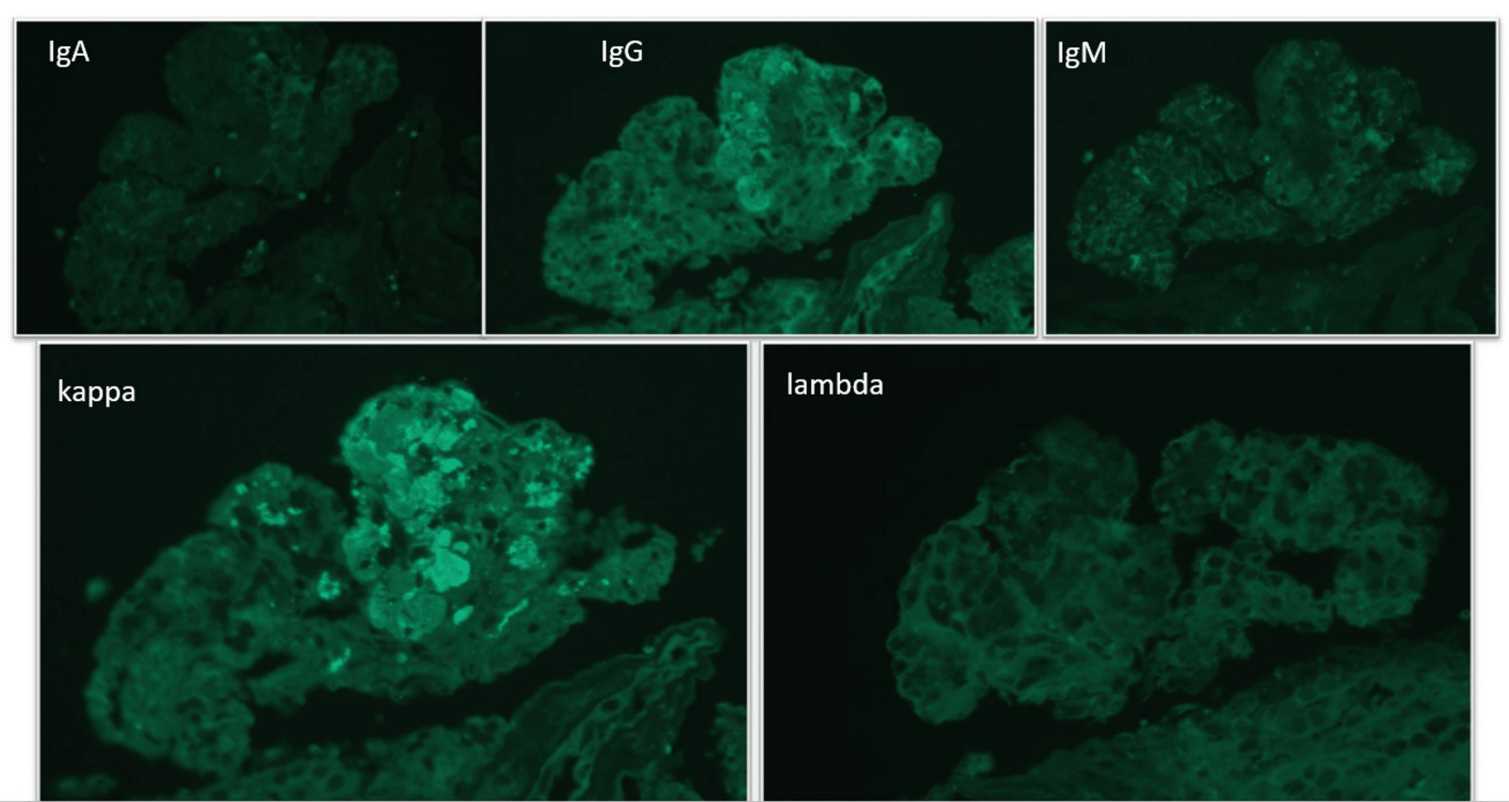
Immunofluorescence (IF) on proteinase-treated paraffin sections: The pseudothrombi in glomerular capillaries show immunoreactivity for IgG (2-3+) and kappa LC (3-4+).

Immunofluorescence (IF) on frozen tissue shows fine granular reactivity for IgG (1 to 2+), kappa LC (1+) and lambda LC (trace to 1+) both along the capillaries and in the mesangium. Reactivity for IgG subtypes reveals IgG2 restriction. There is no significant reactivity for C3 and C1q in the glomeruli. Tubules contain intraluminal casts reactive for polyclonal IgA. The intraluminal casts reveal IgG2 restriction as well. There is stronger kappa light chain reactivity in the background compared to lambda. We also performed immunofluorescence study on protease-treated paraffin sections. The pseudothrombi in glomerular capillaries and small arteries show immunoreactivity for IgG (2-3+) and kappa LC (3-4+) (Figure 3 and Figure 4).

Electron Microscopy

**Figure 5:**
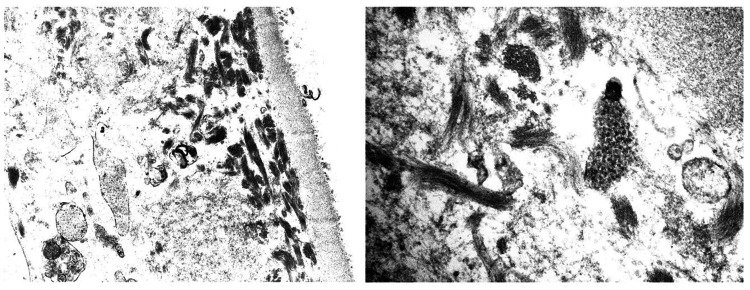
Electron microscopy reveals the presence of subendothelial and few mesangial electron dense deposits. The deposits show curvilinear substructural organization.

Electron microscopy reveals the presence of subendothelial and few mesangial electron-dense deposits. The deposits show curvilinear substructural organization. Double contours of the glomerular basement membranes are formed due to the presence of subendothelial deposits, cellular projections, and new basement membranes under the displaced endothelium (Figure 5).

## Discussion

MGUS, while typically asymptomatic and mostly indolent, may transform to active disease resulting in medical complications, particularly in older adults. It is defined by the presence of a monoclonal protein in the blood, usually detected during routine laboratory work. This condition is relatively common, particularly in individuals over the age of 50, with a prevalence of approximately 3% in this population [4]. The majority of MGUS cases do not progress to malignant diseases, but a small subset (1% annually) may evolve into multiple myeloma, lymphoma, or other plasma cell disorders. This progression is a major concern, particularly when renal involvement occurs, as it may complicate the management and prognosis of these patients [5].

Renal involvement with monoclonal gammopathy may manifest as light chain deposition disease, cast nephropathy, cryoglobulinemia, PGNMID, and C3 glomerulopathy [6]. The mechanism of renal injury in these conditions is typically related to the deposition of monoclonal proteins, which can form aggregates that damage various components of the kidney. Cryoglobulins, which are immune complexes that precipitate in circulation, can cause systemic vasculitis and glomerulonephritis, with significant kidney inflammation and injury [7,8].

Cryoglobulinemic Glomerulonephritis (CGN) is a condition in which cryoglobulins deposit in the kidneys, leading to immune complex-mediated injury. The renal manifestations of CGN may include hematuria, proteinuria, and acute kidney injury leading to chronic kidney disease [9]. CGN is classified into three types, with Type 1 being characterized by the presence of monoclonal immunoglobulin. Types 2 and 3 consist of mixed cryoglobulins that contain both monoclonal and polyclonal immunoglobulins. Type 1 CGN is often associated with hematologic malignancies, including multiple myeloma and Waldenström’s macroglobulinemia [10]. It is typically composed of IgM.

Type I CGN with IgG cryoglobulins, particularly the IgG2‑κ subclass, is extremely rare, with only a single case of IgG2‑κ documented among approximately 10 IgG type I cases in the literature [11]. The presence of IgG cryoglobulins in CGN suggests that these proteins have properties promoting their precipitation and deposition in the renal vasculature. Notably, IgG1 and IgG3 subclasses are strong complement activators via the classical pathway, often leading to significant complement consumption. In contrast, IgG2, such as in our case, is generally a poor activator of complement. The observed low C4 levels may be explained by concurrent involvement of other complement-activating immune complexes or cryoglobulin components, indicating a complex interplay in complement activation beyond IgG subclass alone [12]. IgG2 is a subclass of IgG that has been associated with certain autoimmune conditions, and its presence in cryoglobulinemia may indicate a unique pathophysiologic mechanism underlying renal injury in MGUS [13]. While the exact mechanism of IgG2-kappa cryoglobulin deposition remains unclear, it is likely that factors such as temperature changes, immune dysregulation, and the structural properties of the monoclonal protein contribute to the precipitation and renal deposition of cryoglobulins [14].

Type I CGN is rare and primarily associated with lymphoproliferative diseases. Most studies on it were conducted before widespread HCV testing, and more recent research has focused on cases involving all types of cryoglobulins, particularly HCV-related ones. In a study of 227 patients, 191 showed no clinical signs of CG, while 36 had cryoglobulinemic symptoms. Among these, 25 had IgM cryoglobulins and 11 had IgG. Severe manifestations like skin lesions and nephropathy were more common in patients with IgG cryoglobulins (82%) compared to those with IgM (30%). This highlights the challenges in managing symptomatic Type 1 CG, especially given the lack of long-term data on outcomes [15].

The kidneys are particularly vulnerable to cryoglobulin deposition due to the filtering role of the glomeruli and small vessels. Cryoglobulins can form immune complexes that deposit in the renal vasculature, leading to glomerulonephritis and vasculitis [16].

In addition to MGUS, the present case also has an extended history of rheumatoid arthritis, which may increase the risk of malignant conversion and systemic manifestations. We speculate that symptoms of rash and malignant hypertension were due to vasculitis. Fortunately, he presented to medical care early in the disease process. With timely management, he was able to achieve remission and near-complete renal recovery.

## Conclusions

This case highlights a rare presentation of Type 1 Cryoglobulinemic Glomerulonephritis with IgG2 kappa specificity in a patient with MGUS, emphasizing the potential for monoclonal gammopathies to cause kidney disease. The patient’s recovery following timely diagnosis and treatment underscores the importance of recognizing CGN in MGUS cases. While the patient responded well to corticosteroids and plasmapheresis, the long-term prognosis remains uncertain due to MGUS progression. Further research is needed to elucidate the mechanisms underlying IgG2 kappa cryoglobulin accumulation and to refine treatment strategies for such cases.
